# Macrophage-driven cardiac inflammation and healing: insights from homeostasis and myocardial infarction

**DOI:** 10.1186/s11658-023-00491-4

**Published:** 2023-10-19

**Authors:** Wenjie Zuo, Renhua Sun, Zhenjun Ji, Genshan Ma

**Affiliations:** 1 Department of Cardiology, Zhongda Hospital, School of Medicine, Southeast University, No. 87, Dingjiaqiao, Nanjing, 210009 China; 2 Department of Cardiology, Yancheng No. 1 People’s Hospital, No. 66 South Renmin Road, Yancheng, 224000 China

**Keywords:** Macrophage, Myocardial infarction, Inflammation, Polarization, Hemostasis

## Abstract

Early and prompt reperfusion therapy has markedly improved the survival rates among patients enduring myocardial infarction (MI). Nonetheless, the resulting adverse remodeling and the subsequent onset of heart failure remain formidable clinical management challenges and represent a primary cause of disability in MI patients worldwide. Macrophages play a crucial role in immune system regulation and wield a profound influence over the inflammatory repair process following MI, thereby dictating the degree of myocardial injury and the subsequent pathological remodeling. Despite numerous previous biological studies that established the classical polarization model for macrophages, classifying them as either M1 pro-inflammatory or M2 pro-reparative macrophages, this simplistic categorization falls short of meeting the precision medicine standards, hindering the translational advancement of clinical research. Recently, advances in single-cell sequencing technology have facilitated a more profound exploration of macrophage heterogeneity and plasticity, opening avenues for the development of targeted interventions to address macrophage-related factors in the aftermath of MI. In this review, we provide a summary of macrophage origins, tissue distribution, classification, and surface markers. Furthermore, we delve into the multifaceted roles of macrophages in maintaining cardiac homeostasis and regulating inflammation during the post-MI period.

## Introduction

Myocardial infarction (MI) and its associated heart failure are significant contributors to global mortality and disability, posing a critical threat to public health worldwide [1]. In recent decades, advancements in early reperfusion therapy, particularly the widespread use of direct percutaneous coronary intervention, have revolutionized the emergency management of MI and greatly improved patient survival rates [2]. Nevertheless, in clinical practice, the majority of heart failure cases stem from pathological myocardial remodeling subsequent to MI [3]. Cardiomyocyte death can occur within minutes of ischemia, resulting in cellular ultrastructural changes and increased expression of apoptotic markers [4]. In the acute phase, the necrotic myocardium undergoes progressive shrinkage, leading to thinning of the ventricular wall and reduced mechanical support. Scar formation in this region serves to prevent ventricular aneurysm rupture and further deterioration of cardiac function. While this adaptive remodeling is crucial for preventing early mortality in MI patients, excessive and persistent fibrosis in the infarcted area and surrounding regions can significantly impact ventricular muscle size and function, ultimately culminating in heart failure [5].

The immune response to MI plays a pivotal role in determining the extent of myocardial injury and the severity of subsequent pathological remodeling [6]. Consequently, targeting the inflammatory response has emerged as a crucial approach for cardioprotection [7, 8]. After conducting an extensive review of five large randomized controlled studies, Fiolet et al. [9] reported that low-dose colchicine effectively reduces the risk of major cardiovascular adverse events in patients with coronary heart disease, possibly owing to its broad anti-inflammatory effects. Disruption of the transition from the initial pro-inflammatory response to the subsequent anti-inflammatory repair stage can result in excessive myocardial injury and adverse remodeling, thereby increasing the likelihood of heart failure following MI [10]. However, clinical trials have not provided conclusive evidence regarding the ameliorating effect of immune suppressants on the occurrence and progression of post-MI heart failure, suggesting that the inflammatory response after MI may involve intricate molecular mechanisms and interactions that extend beyond the traditional subgroups and classification of macrophages [11]. Consequently, more precise research tools, such as single-cell sequencing technology, are required to explore the heterogeneity and plasticity of macrophages in greater detail and to develop targeted intervention strategies for the inflammatory response following MI [12, 13]. Considering the crucial involvement of macrophages in immune responses, this review summarizes the origin, tissue distribution, classification, and surface markers of macrophages. Furthermore, we discuss their roles in maintaining cardiac homeostasis and their contributions to post-MI processes, including wound healing, scar formation, and neovascularization.

## Development and classification of macrophages

### Origin and distribution of mononuclear/macrophage system

In the 1960s, van Furth et al. [14] proposed the theory that all tissue macrophages originate from adult mononuclear cells in the peripheral circulation, a viewpoint that has been widely accepted for several decades despite some challenges. Recent studies on cell morphology and function have revealed that the majority of tissue macrophages in adult organisms are already established during embryonic development and derive from the yolk sac [15, 16]. These resident tissue macrophages (RTMs) can exist independently of the peripheral mononuclear system. In fact, each organ possesses its own distinct macrophage system, and RTMs can self-maintain even in individuals with mononuclear cell deficiencies. Embryonic-derived macrophages primarily contribute to tissue remodeling, while those formed during adulthood mainly aid the host in immune defense. These two types of cells coexist in many organs and perform distinct functions in various pathological situations [17]. In contrast, mononuclear cells constitute a highly plastic and dynamic cell system, and their differentiated cells serve as short-lived, immediate-effect executors in the periphery and tissues, facilitating various physiological activities such as vascular neogenesis and immune recognition [18, 19].

On the other hand, various tissues harbor their independent population of RTMs, distinct from peripheral monocytes. These long-lived macrophages play a crucial role in maintaining organ homeostasis by performing essential functions within their respective tissues [20]. RTMs can be classified into different subgroups based on their anatomical location and functional phenotype, including microglia (central nervous system), alveolar macrophages (lungs), osteoclasts (bone), and Kupffer cells (liver) [21–24]. Notably, certain organs like the brain exclusively rely on macrophages derived from embryonic life throughout their lifespan under normal circumstances. However, in other tissues like the heart and intestine, while RTMs possess limited self-renewal capacity, the proportion of embryonic-derived macrophages within the RTM system tends to decline over time. In adults, especially older individuals, peripheral monocytes predominantly contribute to the replenishment of RTMs [25]. Despite recent advances in techniques like lineage tracing that have shed light on RTMs, we still lack a comprehensive understanding of how disease signaling responses differ between embryonic and monocyte-derived RTMs [26].

### Classical polarization theory

The prevailing macrophage polarization model categorizes macrophages into two main subgroups: M1 and M2. Classical activation of M1 macrophages occurs when they receive pro-inflammatory signals, following their differentiation from prototype macrophages. These M1 macrophages exhibit heightened phagocytic and antigen-presenting capabilities, produce reactive oxygen species, and secrete substantial amounts of pro-inflammatory factors and matrix metalloproteinases (MMPs), thereby establishing a local pro-inflammatory microenvironment [27]. In contrast, alternative activation of M2 macrophages takes place through interleukin (IL)-4/IL-13 secretion by type 2 helper T cells. These cells experience upregulation of anti-inflammatory genes and adopt a reparative phenotype, releasing a range of anti-inflammatory cytokines and growth factors. Consequently, M2 macrophages contribute to the production of extracellular matrix (ECM), cell proliferation, and angiogenesis, thereby promoting tissue repair and reconstruction [28]. Additionally, M2 macrophages have been implicated in immune responses, parasite clearance, as well as tumor formation and progression [29]. In light of distinct stimuli that M2 macrophages encounter, they can be further categorized into four subtypes: M2a, M2b, M2c, and M2d [30]. It is important to note that differentiated macrophages have the capacity to undergo "repolarization" in response to pro-inflammatory/anti-inflammatory signals, thereby reversing their phenotype, highlighting the remarkable plasticity of macrophages and their potential as therapeutic agents [31].

### Heterogeneity of macrophages

While the M1/M2 polarization model has been widely employed to depict functional alterations in vitro, the advent of single-cell analysis techniques, such as single-cell ribonucleic acid (RNA) sequencing (scRNA-seq), has rapidly advanced the comprehension of the activation states and intricate phenotypes of macrophages [32]. Traditional RNA sequencing entails examining the entire transcriptome of individual samples, encompassing both messenger RNA and non-coding RNA, to reflect the gene transcription levels within an organism [33]. Nonetheless, even within the same cell type, gene expression at the microscopic level exhibits certain dissimilarities. In contrast, scRNA-seq technology enables the amplification and sequencing of the complete transcriptome of a single cell, facilitating the identification of overall gene expression and distribution information in individual cells [34]. This approach accurately portrays cell heterogeneity and classifies previously rare cell subpopulations, greatly contributing to a deeper understanding of the transcriptional profiles of diverse macrophage populations and their functional disparities in disease models [35]. In terms of the experimental process, scRNA-seq shares similarities with bulk RNA sequencing, involving sample preparation, library construction, and sequencing analysis. However, scRNA-seq incorporates a crucial additional step: the isolation of individual active cells. This can be accomplished through various methods such as flow cytometry, immunomagnetic bead sorting, microfluidics, gradient dilution, among others [36]. During inflammatory conditions, RTMs of embryonic origin are depleted, creating space for circulating monocyte-derived macrophages. These peripheral macrophages, originally pro-inflammatory in nature, undergo a transformation during inflammation resolution and transition into residual RTMs within the tissue, capable of self-renewal. Epigenetic imprints contribute to the distinct labeling of macrophages from different sources, aiding our understanding of why these RTMs exhibit significantly different immune responses despite encountering the same stimulus [37]. Interestingly, due to variations in cytokine gradients within the tissue microenvironment, different groups of macrophages may also experience diverse stimulus conditions, further amplifying the complexity of macrophage phenotypes [38]. Although scRNA-seq appears to elucidate the intricate macrophage types and states in biological tissues, this transcriptome-level analysis falls short in accurately reflecting differences in cell protein function. Additionally, current antibody and mass spectrometry technologies possess inherent limitations. Consequently, the field of single-cell proteomics has emerged, enabling high-throughput analysis of single-cell proteins at an affordable cost through the implementation of automated micro-sample preparation techniques [39]. Using this platform, Specht et al. [40] successfully quantified over 3,042 proteins in 1,490 single nuclei/macrophages within a 10-day timeframe, affirming the substantial heterogeneity exhibited by macrophages in the absence of polarization factors. In summary, the ongoing advancement of single-cell technology presents a novel tool for investigating macrophage heterogeneity, enhancing our comprehension of macrophage dynamics and functions during disease development, and facilitating the creation of single-cell maps that depict macrophage distributions across diverse tissues, contexts, and temporal conditions [41].

## Cardiac macrophages and homeostasis maintenance

### Source and development of cardiac macrophages

**Fig. 1:**
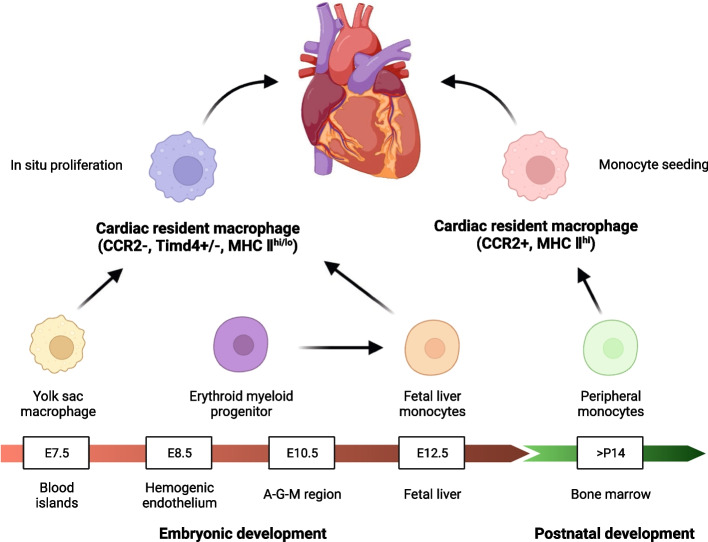
Origin and development of cardiac resident macrophage. During embryonic development, cardiac-resident macrophages primarily originate from the yolk sac or fetal liver and differentiate from monocytes. Notably, monocytes derived from the yolk sac initiate the process of differentiation and migration to the heart earlier, whereas those derived from the fetal liver undergo this process at a later stage. These macrophages derived from embryonic sources colonize the heart and exhibit limited self-replenishment through in situ proliferation. In contrast, bone marrow-derived monocytes start infiltrating the heart at 14 days postpartum and differentiate into an additional major population of cardiac-resident macrophages, primarily supplemented by the continuous colonization of peripheral blood monocytes during later stages. Created with BioRender.com

The heart consists of several major cell types, including cardiomyocytes, cardiac fibroblasts, endothelial cells, and smooth muscle cells. Under homeostatic conditions in mice, cardiac macrophages account for approximately 6–8% of non-cardiomyocytes in the heart [42, 43]. These cardiac resident macrophages (CRMs) exhibit a spindle-shaped morphology and are distributed among cardiomyocytes, endothelial cells, and fibroblasts [43]. Genetic mapping and lineage tracing techniques have confirmed that the majority of CRMs originate from the yolk sac or fetal liver during embryonic development, although this theory has been subject to debate. The formation of CRMs is influenced, at least in part, by these two components [44]. The source and development process of CRMs can be summarized as follows (Fig. 1): CRMs derived from yolk sac macrophages initiate development on day 7 of embryonic development and migrate to the heart on day 7.5. Yolk sac hematopoietic endothelium and erythro-myeloid progenitor cells in the aorta-gonad-mesonephros and fetal liver, respectively, differentiate into fetal liver monocytes on day 8.5 and day 10.5 after development and migration. This group of cells predominantly forms on day 12.5 and eventually merges with the former, primarily representing CC chemokine receptor 2 (CCR2)^−^ CRMs. Local proliferation mainly supplements this subgroup [45]. In contrast, peripheral bone marrow monocytes undergo transformation into CCR2^+^ CRMs after 14 days of self-production. This subgroup is supplemented through recruitment from circulating monocytes and local proliferation [44]. CRMs undergo self-renewal through cell proliferation approximately once a month within the heart. Initially derived from embryos, CRMs possess similar cellular characteristics but exhibit different phenotypes and functions upon settling in unique tissue microenvironments. They play a crucial role in maintaining homeostasis, heart development, and immune surveillance in cardiac tissues. However, their precise involvement in cardiac inflammation requires further investigation [44, 46]. As mentioned earlier, although CRMs have a longer lifespan and can self-renew, the decline in their ability to divide with age or their consumption in pathological conditions poses challenges in maintaining the original pool of CRM cells. Therefore, recruitment of peripheral circulating monocytes is necessary to replenish them [44, 46].

### Phenotype of cardiac macrophages

In steady-state conditions, the inherent CRMs exhibit significant heterogeneity due to their diverse origins. Currently, the commonly employed markers are major histocompatibility complex II (MHC II) and CCR2. Based on the expression levels of these markers, CRMs are roughly categorized into four subgroups: CCR2^+^MHC II^lo^, CCR2^+^MHC II^hi^, CCR2^−^MHC II^lo^, and CCR2^−^MHC II^hi^ macrophages [47, 48]. CCR2, serving as a receptor for monocyte chemoattractant protein, primarily facilitates monocyte infiltration into tissues during inflammation, and its expression level indicates the origin of CRMs from either peripheral monocytes or embryonic progenitor cells [43, 46]. Recent studies have revealed distinct roles of CCR2^+^ and CCR2^−^ macrophages in heart repair after injury [49, 50]. MHC II plays a role in macrophages presenting antigens to T lymphocytes and is associated with the activation of adaptive immunity. Macrophages with high MHC II expression more effectively present antigens to T lymphocytes, while those with low MHC II expression exhibit stronger phagocytic ability [51]. T-cell immunoglobulin and mucin domain-containing protein 4 (Timd4) serve as another surface marker that distinguishes embryonic and monocyte-derived CRMs, as it is highly expressed in embryonic-derived CRMs but not in monocyte-derived CRMs [52]. Additionally, Ly6C, CX3CR1, and CD11c are commonly employed markers for distinguishing mouse CRMs [53]. Apart from surface markers, scRNA-seq is increasingly used to identify CRM heterogeneity and provide more detailed information [36]. Dick et al. [52] conducted scRNA-seq on CRMs isolated from healthy, non-injured mice, resulting in the reclassification of steady-state CRMs into four subtypes: 1) CRMs with high expression of Timd4, Folr2, Lyve1, CD163, and Igf1; 2) CRMs with high expression of MHC II, F4/80, CX3CR1, and CD14; 3) CRMs with high expression of CCR2 and CD64; and 4) CRMs with high expression of Ifit1, Ifit3, and Irf7. Bioinformatics analysis revealed that subtypes 3 and 4 are upregulated in the classical inflammatory pathway and closely associated with peripheral monocytes, subtype 2 primarily relates to translation, ribosomes, and antigen presentation, and subtype 1 exhibits higher vascular neogenesis, endocytosis, and lysosomal activity.

### The effect of cardiac macrophages on homeostasis

In a healthy state, CRMs act as M2 repair-type macrophages, expressing anti-inflammatory genes and promoting tissue reconstruction after injury, thereby maintaining homeostasis [54]. CCR2^+^ macrophages derived from the yolk sac are crucial for coronary artery development and cardiac repair, as their absence during embryonic development can lead to abnormal cardiac remodeling, left ventricular morphology, and functional disorders [55]. These macrophages complement circulating CCR2^+^ macrophages, which can rapidly express pro-inflammatory genes, to maintain cardiac homeostasis. CRMs are essential for maintaining cardiac physiological homeostasis in three ways: capillary formation and lymphatic network maturation, cardiac electrical signal conduction, and mitochondrial function regulation [15].

Located in the myocardium near the epicardium, CCR2^+^ macrophages from the yolk sac are closely related to the formation and functional maturation of the coronary vascular system. They mediate the development of coronary arteries by secreting insulin-like growth factor 1, inducing capillary formation and endothelial cell migration [55]. In mouse strains lacking these macrophages, the vascular system in the heart exhibits an abnormal distribution pattern, characterized by an increase in the number of small-diameter capillaries and a decrease in the number of large-diameter capillaries, and excessive branching of blood vessels, making it more difficult for blood to perfuse into the cardiac microcirculation system [55]. Furthermore, Lyve1-positive CRMs from the yolk sac can act as companion cells, guiding lymphatic endothelial cells to come closer together to form lymph nodes, thus constructing the lymphatic network in the heart. These CRMs are adjacent to endothelial cells and have high expression of genes related to angiogenesis and endothelial tube formation. Lack of CRMs during early embryonic development inhibits lymphatic vessel formation [56, 57].

In both humans and mice, the atrioventricular junctions contain CCR2^+^ and CCR2^−^ CRM-expressing cells that establish connections with cardiomyocytes through gap junctions, leading to synchronized depolarization. Depletion of these CRMs or loss of connexin 43 (Cx43), a key component of the gap junctions, results in atrioventricular conduction block and subsequent atrioventricular block [58]. One possible mechanism is that the presence of these CRM-cardiomyocyte connections reduces the electrical refractory period and enhances heart conduction [58]. To investigate this mechanism, diphtheria toxin was used to deplete the existing CRMs in mice, which led to spontaneous atrioventricular block, despite monocytes from the periphery filling the gap and proliferating again [59, 60]. Depletion of other immune cells such as lymphocytes and granulocytes, followed by inducing stress in mice hearts through pulmonary artery ligation, did not result in severe atrioventricular block or sudden death. This process may be attributed to CRMs secreting the bimodal protein amphiregulin, which promotes the phosphorylation of Cx43 in cardiomyocytes [61].

Furthermore, CRMs expressing c-mer tyrosine kinase (Mertk), CD206, and other receptors are widely distributed around cardiomyocytes, suggesting their potential role in clearing extracellular vesicles, including damaged mitochondria and other organelles, released through cell exocytosis [53, 62]. These extracellular vesicles are typically generated and secreted during the process of autophagy in cardiomyocytes, and the active uptake and ingestion of this cellular waste by CRMs are crucial for maintaining the extracellular environment and normal cardiac function [63]. Depletion of CRMs or knockout of the Mertk receptor leads to a significant increase in extracellular vesicle numbers in the cardiomyocytes' extracellular environment, accumulation of intracellular debris, and impairment of the heart's basic aerobic metabolic function. This, in turn, affects the heart's normal contraction and relaxation, representing an important mechanism for stress-induced damage to the heart [62, 64]. Additionally, these CRMs expressing the Mertk receptor can provide partial protection against arrhythmias induced by infection in young mice, showcasing their role in immune surveillance [65].

## Macrophages in the development of MI

### Atherosclerosis

Macrophages play a dual role in the occurrence and development of atherosclerosis. In the early stages of the disease, foam cells are capable of engulfing and clearing apoptotic cells at the lesion site, thus impeding lesion progression. However, during late-stage lesions, the impaired phagocytic function of macrophages hinders the effective clearance of these apoptotic fragments, facilitating the formation of necrotic cores beneath the endothelium. Consequently, macrophages transition to a pro-inflammatory phenotype, further intensifying inflammation and contributing to atherosclerotic plaque formation [66, 67]. Additionally, macrophage-secreted MMPs may contribute to thinning the fibrous cap, which can lead to plaque rupture and thrombus formation [68]. Furthermore, macrophages also participate in the process of plaque regression. In mouse models exposed to high-fat or high-sugar diets, plaque regression can be induced by actively reducing blood lipid or glucose levels. This regression is characterized by a reduction in macrophages within the lesion area and alterations in the gene expression of the remaining CD68-positive cells. Macrophages in regressing lesions with CD68 positivity exhibit a predominantly M2 phenotype, as evidenced by increased expression of Arg1 and CD163, as well as decreased expression of pro-inflammatory factors CCL2 and TNF-α [69].

However, the heterogeneity of macrophages plays a crucial role in the complex process of atherosclerosis, and the simplistic in vitro classification of M1/M2 polarization fails to fully capture their diverse functions. Throughout the study of atherosclerosis, several additional macrophage subtypes have been progressively identified. Among these subtypes, hemorrhagic Mhem macrophages constitute a distinct group that specializes in phagocytizing and utilizing residual components of red blood cells, specifically hemoglobin. By utilizing various pathways such as ATP-binding cassette transporter A1 and G1, as well as the nuclear liver X receptor, these macrophages hinder foam cell formation and decelerate the progression of atherosclerosis [70, 71]. Mox macrophages, on the other hand, arise from the induction of M0 macrophages with oxidized phospholipids. These macrophages regulate antioxidant enzymes through the nuclear factor-related factor 2, thereby mitigating oxidative stress damage. Within the lesions of LDL receptor knockout mice, Mox macrophages are widely distributed and account for approximately 30% of the total macrophage population [72]. Furthermore, the chemokine ligand CXCL4 inhibits CD163 and atheroprotective enzyme heme oxygenase-1 in macrophages, compromising their phagocytic function and exacerbating the progression of atherosclerosis [73].

scRNA-seq analysis conducted in the mouse models of atherosclerosis indicates that macrophages and T cells are the predominant types of white blood cells found in the plaque, with the proportion of macrophages increasing as the plaque progresses [74, 75]. There are three primary types of macrophages within plaques: resident macrophages, pro-inflammatory macrophages, and foam macrophages characterized by high triggering receptor expressed on myeloid cells 2 (Trem2) expression. Resident macrophages derived from healthy aortas primarily express CX3CR1 and Lyve 1, displaying M2-type characteristics with elevated expression of anti-inflammatory markers. Depletion of these cells accelerates atherosclerosis and collagen deposition [76–78]. However, in resident macrophages of atherosclerotic aortas, CCR2 is primarily expressed, indicating that during pathological progression, embryonic-origin macrophages are gradually replaced by peripheral-origin macrophages, which mainly express M2 anti-inflammatory markers such as Folr2, Cbr2, Pf4, and CD206 [76, 79]. Monocytes recruited to the vascular intima via chemotaxis can differentiate into pro-inflammatory macrophages, expressing surface molecules such as MHC II, CD64, CD80, and releasing inducible nitric oxide synthase (iNOS), IL-6, and TNF-α [80]. These pro-inflammatory macrophages constitute nearly half of the macrophage subpopulation, primarily comprising non-foam-like macrophages, and play a pivotal role in driving the entire inflammatory lesion [79]. Trem2, a bone marrow-specific transmembrane protein, has been shown in previous studies to be inversely correlated with plaque stability [81]. It should be noted that Trem2 is not detectable in macrophages of healthy aortas and is mainly present in atherosclerotic lesions [74]. In Trem2^hi^ macrophages, upregulation of CD9, Spp1, Hvcn1, and several tissue proteases occurs, which can increase the vulnerability of atherosclerotic inflammation and plaque damage [82]. These foam-like lipid-laden macrophages exist in the early and late stages of the lesion, accumulating in the intima and necrotic core of the plaque, although their precise phenotype remains unclear. Therefore, future investigations should employ next-generation technologies to generate more comprehensive transcriptome or proteome maps of mouse and human macrophages, aiding in the elucidation of the specific roles of macrophage subgroups in different stages of atherosclerosis.

### Inflammation response and resolution

**Fig. 2:**
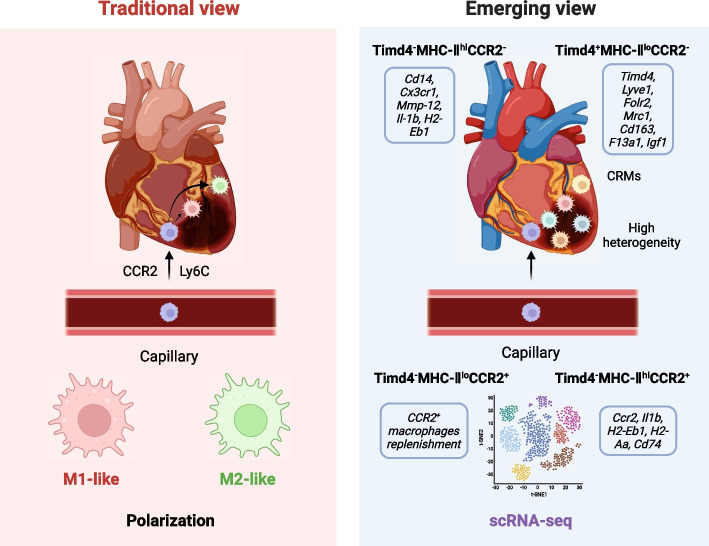
Advancements in the understanding of macrophage populations and functions after MI. According to the traditional view, macrophages were classified into two populations: pro-inflammatory M1 macrophages and reparative M2 macrophages. Initial investigations into cardiac macrophage populations after myocardial infarction (MI) seemed to support this established model. However, recent evidence, not only from heart but also from other tissues, has unveiled a remarkable heterogeneity and plasticity in macrophage development, phenotype, and function. The introduction of single-cell RNA sequencing has significantly advanced the understanding of distinct macrophage clusters and their phenotypes after MI. These emerging findings in macrophage biology have provided insight into the shortcomings of non-specific immunosuppressive strategies and have paved the way for novel therapeutic interventions targeting heart failure prevention following MI. Adapted from “Healthy vs. Diseased Heart (Layout)”, by BioRender.com (2020). Retrieved from https://app.biorender.com/biorender-templates

Due to the limited regenerative capacity of adult mammalian hearts, appropriate inflammation and repair following MI are crucial for maintaining the structural and functional integrity of the heart [83]. The post-MI response encompasses three temporally continuous and overlapping stages: inflammation, scar formation, and tissue remodeling. Macrophages, serving as both effector and regulator, play vital roles in these stages. Previous studies have demonstrated that early depletion of monocyte-derived macrophages or inhibition of macrophage migration function leads to inadequate repair after MI and pathological ventricular remodeling [84]. Several theories exist regarding the phenotypic changes of macrophages following MI. After myocardial cell death, neutrophils and CRMs regulate the infiltration of damaged myocardial tissue by a large number of circulated Ly6C^hi^ monocytes. CCR2^+^ CRMs promote monocyte recruitment, release of monocyte chemoattractant proteins, and monocyte mobilization through the MYD88 pathway. Conversely, CCR2^−^ CRMs inhibit monocyte recruitment [47]. Ly6C^hi^ monocytes replace the initial neutrophils in the days following MI and become the predominant immune cell population in the damaged area, peaking on the third day after MI [85]. Peripheral monocytes differentiate into M1-like macrophages, which phagocytize cell debris, degrade ECM, and stimulate the inflammatory response by secreting TNF-α, IL-1β, and IL-6. This initiates the repair mechanism after MI, but excessive inflammatory responses can cause secondary damage to the infarcted myocardium and worsen myocardial remodeling [86]. From day 4 to 7 after MI, Ly6C^lo^ monocytes are preferentially recruited to the myocardium and differentiate into M2-like macrophages, which facilitate ECM remodeling and angiogenesis through the secretion of anti-inflammatory factors such as IL-10 [86]. However, categorizing macrophages strictly as M1 or M2 may oversimplify their functional diversity, as macrophages possess plasticity and can dynamically change their surface markers over time and space (Fig. 2) [87]. Mouton et al. [88] isolated macrophages from the infarct area of mice on days 1, 3, and 7 after MI and analyzed their transcriptome characteristics. Although F4/80^lo^Ly6C^hi^ monocytes exhibited a significant pro-inflammatory phenotype on day 1, and macrophages displayed clear repair features on day 7, both cell types did not fully align with the M1/M2 phenotypes, such as the overexpression of M2 markers Arg1 by pro-inflammatory macrophages on day 1. Macrophages on day 3 exhibited enhanced proliferation and phagocytic function, along with gene expression related to mitochondrial function and oxidative phosphorylation, underscoring the significance of metabolic reprogramming in macrophage polarization. In summary, macrophages play critical roles in both healthy hearts and damaged myocardium following MI, and their functions and phenotypes are complex and dynamic.

In recent years, the development of scRNA-seq technology has provided us with a deeper understanding of the macrophages after MI. The major subpopulations of macrophages involved in cardiac homeostasis and MI are shown in Table 1. Bajpai et al. [47] demonstrated the distinct functions of CCR2^+^ and CCR2^−^ CRMs following MI. Depletion of selective CRMs in the infarct border region impacts monocyte recruitment and the accumulation of phagocytic macrophages, resulting in impaired healing and adverse remodeling. Dick et al. [52] observed a wide range of macrophage diversity in the heart following ischemic injury, with Timd4 and CCR2 acting as CRMs and recruitment markers. The number of CRMs in the infarcted area initially decreases significantly but gradually replenishes through self-renewal. Jin et al. [89] discovered that macrophages are the predominant CD45^+^ immune cell population in the heart of a mouse model with MI induced by permanent left anterior descending coronary artery ligation. The macrophage population reaches its peak on the third day after MI. Inhibiting the prevailing inflammatory subgroup during this period can prevent subsequent leukocyte extravasation and adverse remodeling. Furthermore, on the 7th and 14th day following MI, a substantial presence of apoptotic neutrophils and pro-fibrotic macrophage subgroups was observed. Zhuang et al. [90] identified two ischemia-related macrophage clusters: Olr1-rich macrophages exhibit predominantly pro-inflammatory characteristics, while Gpnmb-rich macrophages are more inclined towards phagocytosis and fatty acid oxidation. Rizzo et al. [91] also characterized the temporal expression profile of macrophages in MI mice. Macrophages in the infarct area predominantly originated from peripheral monocytes, comprising two pro-inflammatory subgroups exhibiting Isg15^hi^ and MHCII^+^Il1b^+^ phenotypes, as well as non-inflammatory Trem2^hi^ cells. Ischemic injury triggers the conversion of circulating Ly6C^hi^ monocytes into Chil3^hi^ states exhibiting granulocyte characteristics, whereas ischemic tissues display Trem2^hi^ macrophages with similarities to foam macrophages observed in atherosclerotic mouse models. Jung et al. [92] developed a spatial–temporal transcriptional map of macrophages following MI, revealing that macrophages constituted over 80% of immune cells at nearly all time points post-MI. During the late stage of MI (days 5–7), there was an up-regulation of Trem2 expression in macrophages, and the administration of soluble Trem2 in the animal model substantially enhanced the structure and function of the infarcted heart, indicating the therapeutic promise of this signaling pathway for the future. A better understanding of the similarities and differences between human and animal macrophages will facilitate the implementation of clinical translational research. Similar to mice, cardiac CD45^+^CD64^+^CD14^+^ macrophages in patients with cardiomyopathy can also be categorized based on CCR2 and MHC-II (HLA-DR2) expression: CCR2^−^MHC-II^hi^, CCR2^+^MHC-II^hi^, and CCR2^+^MHC-II^lo^ subsets [52]. CCR2^+^MHC-II^hi^ macrophages are maintained by peripheral monocyte replenishment and limited self-renewal while CCR2^−^MHC-II^hi^ macrophages rely only on proliferation in situ. Although there are slight differences in the transcripts expressed by macrophages between humans and mice, CCR2 is used in both to define monocytes and monocyte-derived macrophages recruited to the myocardium. Interestingly, the percentage and abundance of CCR2^+^ macrophages were positively associated with systolic dysfunction in patients with heart failure [49]. The spatial location of macrophages is also closely related to their function. CCR2^−^MHC-II^hi^ macrophages are primarily located near vessels in normal, uninjured myocardium and expressed anti-inflammatory genes while CCR2^+^MHC-II^hi^ subset accumulates in scar tissue and mediates the classical inflammatory pathways [49].

**Table 1:** Major subpopulations of macrophages involved in steady state and myocardial infarction

Clusters	Origin	Gene transcripts	Lifecycle	Phenotype activity
Cardiac resident macrophages				
Timd4^+^MHC-II^lo^CCR2^−^	Embryonic development	Timd4, Lyve1, Folr2, Mrc1, cd163, F13a1, Igf1	In situ proliferation	• Homeostasis • Endocytosis • Angiogenesis • Lymphatic development
Timd4^−^MHC-II^hi^CCR2^−^	Embryonic development	Cd14, Cx3cr1, Mmp-12, IL1b, H2-Eb1	In situ proliferation & monocyte replenishment	• Hemostasis • Antigen presentation
Timd4^−^MHC-II^hi^CCR2^+^	Peripheral circulation	Ccr2, IL1b, H2-Eb1, H2-Aa, Cd74	Monocyte replenishment	• Classical pro-inflammatory pathways • Post-MI chemotaxis
Monocytes				
Timd4^−^MHC-II^lo^CCR2^+^	Peripheral circulation	Unknown	Monocyte infiltration	• CCR2^+^ macrophage replenishment

*Timd4* T-cell immunoglobulin and mucin domain-containing protein 4, *MHC-II* major histocompatibility complex II, *CCR2* C–C chemokine motif receptor 2, *Lyve1* lymphatic vessel endothelial receptor 1, *Folr2* folate binding protein-2 receptor, *Mrc1* mannose receptor C-type 1, *F13a1* coagulation factor 13 subunit A, *Igf1* insulin-like growth factor 1, *Cx3cr1* C-X3-C motif chemokine receptor 1, *Mmp-12* matrix metalloproteinase 12, *IL1b* interleukin-1 beta, *H2-Eb1* histocompatibility 2 class II antigen E beta, *H2-Aa* histocompatibility 2 class II antigen A alpha, *MI* myocardial infarction

Not only do macrophages mediate the inflammatory response after MI, but they also synthesize and release pro-resolving signals. Through lipoxygenase-mediated maresin activation, they collaborate with neutrophils and platelets to clear dead cells and inflammation in the infarcted area [93]. After MI, splenic leukocytes synthesize a large number of pro-resolving mediators and carry them to infiltrate the left ventricle. Macrophages are essential for the synthesis of these mediators, and their abundance in synthesis determines the time for inflammation resolution in infarcted myocardium [94]. Considering that inhibiting post-MI inflammation in early clinical trials has not yielded ideal results, regulating macrophages to promote the resolution of myocardial inflammation may be a promising treatment strategy in the future. Jia et al. [95] identified legumain as a gene specifically expressed in CRMs, and the absence of legumain impairs the inflammation resolution and phagocytic clearance function mediated by CRMs after MI, leading to a significant deterioration in cardiac function. Rienks et al. [96] found that Semaphorin3A expression increased in MI patients 30 days after admission. In mice, Semaphorin3A can promote apoptosis in pro-inflammatory macrophages and polarize them toward the resolution phase, delaying the migration of monocytes to the myocardium and improving cardiac function. Macrophages receive signals from other cell sources, influencing inflammation resolution. The specific loss of endothelial cell sphingosine 1-phosphate receptor 1 significantly reduces the accumulation of Ly6C^lo^ macrophages after MI [97]. Foxp3^+^CD4^+^ regulatory T cells induce M2 differentiation of macrophages within the myocardium, assisting in inflammation resolution [98]. Moreover, disruptions in the synthesis of bioactive metabolites can affect macrophage-mediated inflammation resolution. For instance, 12/15-lipoxygenase-deficient mice exhibit changes in lipid synthesis and metabolomics after MI, lower levels of pro-inflammatory metabolites, and this metabolic reprogramming promotes effector leukocytes, encourages M2 polarization, suppresses excessive collagen deposition, ultimately slowing the progression of heart failure, and increasing survival [99, 100]. In contrast, arachidonate 5-lipoxygenase contributes to the endogenous synthesis of pro-resolving mediators, which are crucial for leukocyte clearance of dead myocardial cells, inflammation regulation, and differentiation of cardiac fibroblasts. It coordinates the start of the resolution phase, promoting effective healing of myocardial tissue after MI [101]. Interestingly, sex and aging can influence the synthesis of inflammatory factors after MI and macrophage responses. Female mice in an acute MI-induced heart failure model exhibit increased survival rates, improved cardiac function, limited adverse remodeling, and lower abundance of inflammatory factor synthesis compared to male mice, indicating a milder inflammatory response [102]. In young mice, levels of pro-inflammatory metabolites derived from arachidonic acid are significantly higher than in older mice after MI, despite no significant differences in infarct size and cardiac function between the two groups. Older mice with excessive intake of fatty acids exhibit increased pro-inflammatory Ly6C^hi^ macrophages, elevated levels of inflammatory factors, and increased renal damage [103]. Furthermore, the potential impact of medication on macrophage responses is worth noting. Doxorubicin primarily exhibits cardiac toxicity characterized by immune metabolic dysfunction. It induces splenic germinal center contraction and reduces CD169^+^ macrophages, impairing the resolution of inflammation [104]. Subacute injection of the nonsteroidal anti-inflammatory drug carprofen affects neutrophil phagocytic function and reduces reparative macrophages in mice after MI, causing delayed resolution of inflammation [105]. Formyl peptide receptor 2 (FPR2), as a G protein-coupled receptor, plays an important role in immune cell recruitment after injury [106]. Using the selective agonist BMS-986235 enhances phagocytic activity and neutrophil apoptosis in an MI animal model. By regulating macrophage polarization, this molecule preserves viable myocardium, attenuates adverse left ventricular remodeling, and maintains cardiac function [107]. In contrast, injecting the FPR2 antagonist WRW4 induces the recruitment of immature leukocytes and increases the number of pro-inflammatory macrophages, disrupting the resolution of inflammation after MI [108].

### Trauma healing and scar formation

Following MI, one of the primary functions of macrophages is to phagocytose apoptotic and necrotic cells within the infarct region. Phagocytic macrophages respond to initial signals from neutrophils and cell debris and are guided to the site of injury by "find me" signals. This process is coordinated by receptors including efferocytosis receptors, integrin receptors, and complement receptor 3. Notably, the Mertk receptor is essential for the clearance of necrotic myocardial cells. Following MI, Mertk is specifically expressed in repair type M2 macrophages with low Ly6C levels, contributing to the resolution of inflammation. Conversely, the absence of Mertk in macrophages leads to a specific reduction in their ability to phagocytose myocardial cells, thereby impacting the subsequent myocardial repair process [109]. Galectin-3 is a widely expressed molecule in immune cells that plays a role in immune regulation. Both in vivo and in vitro studies have demonstrated that the deficiency of this molecule impairs the phagocytic activity of macrophages towards apoptotic cells [110]. Cassaglia et al. [111] further confirmed that the absence of this molecule significantly impacts macrophage infiltration, scar formation, and fibrosis following MI. This finding suggests a direct association between the clearance of damaged regions by macrophages in the early stages of MI and subsequent ventricular remodeling. Cai et al. [112] discovered that mitochondrial dysfunction in macrophages exacerbates post-MI inflammation and hampers subsequent normal repair processes, which are originally facilitated through macrophage efferocytosis and intercellular communication between macrophages and fibroblasts. Notably, Smad3-specific knockout mice in the myeloid lineage exhibited heightened late-stage mortality and myocardial remodeling after MI, attributed to deficiencies in macrophage phagocytic capacity, impaired anti-inflammatory phenotype switching, uncontrolled scar expansion, and excessive myocardial cell apoptosis. Furthermore, in vitro experiments demonstrated that Smad3-deficient macrophages displayed reduced "eat me" signals and anti-inflammatory gene expression [113]. Interestingly, the addition of apoptotic cells to macrophages in vitro induced the secretion of vascular endothelial growth factor (VEGF), while in vivo, mice with defective efferocytosis exhibited diminished lymphatic vessel neogenesis and VEGF levels after MI, providing additional evidence from a different perspective to support the correlation between macrophage efferocytosis function and subsequent myocardial repair and remodeling following MI [114]. Additionally, Lindsey et al. [115] observed a significant increase in CXCL4 levels in the infarct area on the third day after MI. Intravenous infusion of CXCL4, compared to saline, notably induced cardiac volume expansion and heightened 7-day mortality after MI. In vitro experiments suggested that CXCL4 inhibits CD36 signals, thereby diminishing macrophage phagocytic capacity.

After post-MI acute inflammation, the subsequent stages involve the participation of pro-reparative macrophages and cardiac fibroblasts. These cellular components play a crucial role in facilitating adaptive remodeling of the myocardium and scar formation, thereby maintaining normal cardiac function. The newly formed connective tissue, developing blood vessels, and ongoing scar formation collectively constitute the granulation tissue, which replaces the damaged tissue cleared by macrophages, ultimately completing the process of wound healing. In response to MI, cardiac fibroblasts actively engage in multiple aspects related to the myocardium, including cardiac structure, mechanical and electrical activities, as well as biochemistry. A pivotal step in the fibrosis process is the transformation of fibroblasts into myofibroblasts, characterized by increased expression of α-smooth muscle actin, enhanced production of ECM components (such as collagen I, collagen III, fibronectin, and laminin), and secretion of factors involved in myocardial remodeling, including transforming growth factor-β (TGF-β), MMPs, angiotensin II, and endothelin I [116]. Macrophages, on the other hand, contribute to this differentiation process by secreting inflammatory mediators like TGF-β, IL-10, and CCL17. These mediators enhance ECM synthesis and inhibit its degradation, thereby promoting tissue remodeling and repair following MI. Jung et al. [117] demonstrated that compared to the control group of mice, in vitro infusion of IL-10 increased the number of anti-inflammatory macrophages, stimulated proliferation and migration of cardiac fibroblasts, and effectively regulated collagen distribution within the myocardial walls. This regulation resulted in improved ventricular compliance and enhanced cardiac function. Emerging evidence suggests that macrophages may directly participate in myocardial fibrosis and scar formation by secreting ECM proteins. Within the first day after MI, macrophages can transform into fibroblast-like cells and upregulate fibroblast-specific ECM genes, such as Col7a16 and Postn. However, these cells lack fibroblast markers, such as DDR32 [118]. In a mouse model of chronic renal injury, fibroblast-like macrophages were found to promote interstitial fibrosis in the kidney and were associated with transplant rejection [119]. Therefore, macrophages may be involved in the fibrosis process after MI through direct or indirect pathways. Future research could focus on utilizing techniques like scRNA-seq to determine the functions of distinct cell subpopulations. This approach would help gain a better understanding of the effects of immune cell-mediated ECM synthesis on scar characteristics and identify the optimal balance between immunity and fibrosis.

### Immunometabolism in hypoxic microenvironment of MI

**Fig. 3:**
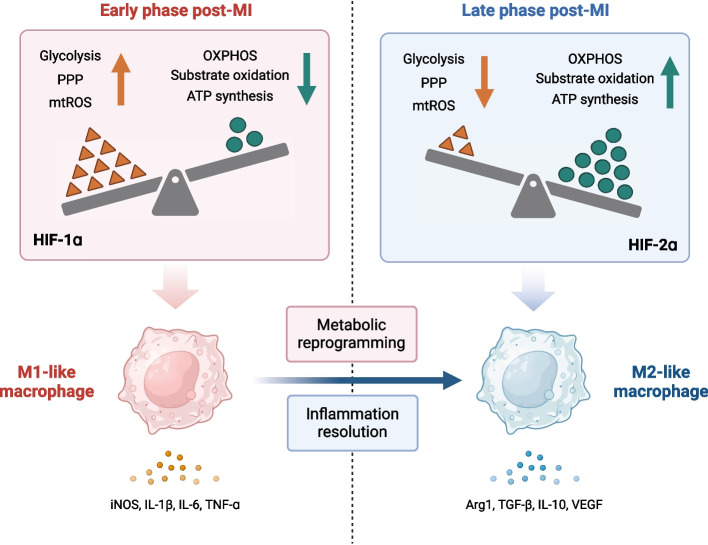
Metabolic reprogramming of macrophages after MI. In the early phase post myocardial infarction (MI), macrophages infiltrate the injured myocardium and secrete pro-inflammatory cytokines. Under hypoxic stimuli, there is a shift in their energy metabolism, favoring glycolysis through the pentose phosphate pathway (PPP). Additionally, there is rewiring of the tricarboxylic acid cycle (TCA) and an elevation in mitochondrial reactive oxygen species (mtROS) levels. As a result, substrate oxidation and adenosine triphosphate (ATP) synthesis via oxidative phosphorylation (OXPHOS) are reduced. In contrast, macrophages adopt a phagocytotic phenotype during the wound healing phase. This transition is driven by changes in the microenvironment, efferocytosis (the clearance of apoptotic cells), and further metabolic reprogramming. Consequently, macrophages switch back to oxidative metabolism and actively participate in collagen deposition, promoting tissue repair and healing processes. The balance between hypoxia-induced factor-1ɑ (HIF-1ɑ) and HIF-2ɑ is also involved in the metabolic reprogramming and phenotype switching of macrophages. HIF-1ɑ induces the conversion of I-arginine into inducible nitric oxide synthase (iNOS) while HIF-2 induces the production of arginase-1. Adapted from “Comparison Between Oxidative Eustress and Oxidative Distress”, by BioRender.com (2020). Retrieved from https://app.biorender.com/biorender-templates

Hypoxia is a hallmark of MI, and the continued infiltration of immune cells further exacerbates the local oxygen depletion in the infarcted area. In such a hypoxic microenvironment, macrophages will undergo a metabolic shift, primarily relying on oxygen-independent glycolysis and rapidly accumulating hypoxia-inducible factors (HIFs) within the cell nucleus. Extensive research has confirmed the close association between macrophage responses to hypoxic stimuli, metabolic reprogramming, and downstream functional phenotypes. In the early stage of MI, pro-inflammatory macrophages exhibit a tendency toward glycolysis and the pentose phosphate pathway (PPP), accompanied by increased levels of the tricarboxylic acid (TCA) cycle and mitochondrial reactive oxidative stress [120]. HIF-1α appears to be a fundamental component of this process, as it enhances the expression of kinases downstream of glycolysis and prevents glucose entry into the TCA cycle, reducing oxidative phosphorylation rates [121]. In contrast, in the late stage of MI, pro-reparative macrophages favor oxidative phosphorylation, with increased substrate oxidation, fatty acid uptake, and adenosine triphosphate (ATP) synthesis (Fig. 3) [122]. This metabolic mode provides them with a longer-lasting energy supply, consistent with their function. Pro-reparative macrophages appear later and primarily engage in efferocytosis, collagen deposition, and ECM synthesis. Glycolytic metabolism analysis also corroborated this, as gene expression related to glycolysis increased 1 day after MI, TCA gene expression increased on day 3 and day 7 after MI, and PPP gene expression also increased on day 7 after MI, whereas this metabolic reprogramming phenomenon is observed in monocyte-derived macrophages rather than RTMs [123].

Following myocardial ischemia, the initial regional distribution of HIF-1α and HIF-2α is similar, primarily present at the infarct border, while HIF-2α increases predominantly in the remote infarct area later [124]. HIF-2α inhibits macrophage mitochondrial metabolism, while HIF-1α, through glycolytic reprogramming, cleaves the cardioprotective Mertk receptor. However, the combined absence of both subtypes in myeloid cells leads to macrophage necrosis, fibrosis, and cardiac rupture [125]. It seems that HIF-1α and HIF-2α complement rather than act individually. In vitro experiments also confirm this, where HIF-1α is mainly expressed in pro-inflammatory macrophages, promoting iNOS expression and nitric oxide synthesis, while HIF-2α is predominantly expressed in pro-reparative macrophages, promoting arg-1 expression and inhibiting nitric oxide synthesis. The HIF-α isoform switch is of significant importance for macrophage phenotypic transition and metabolic reprogramming after hypoxia, and many molecules exert their effects by influencing HIF-related pathways. It's worth noting that pyruvate kinase M2 (PKM2), as a key enzyme in glycolysis, can form a complex with HIF-1α under lipopolysaccharide stimulation, directly binding to the IL-1β promoter, stimulating the formation of pro-inflammatory macrophage phenotypes, namely the Warburg effect. Knocking down PKM2 can inhibit this effect, reversing macrophage transition to a reparative phenotype and reducing the release of high mobility group box-1 [126, 127]. Cheng et al. [128] found that ω-Alkynyl arachidonic acid inhibits overexpression and nuclear translocation of PKM2 in macrophages under lipopolysaccharide stimulation, disrupting its binding with HIF-1α, thereby reducing post-MI inflammation levels and infarct size. Lu et al. [129] also discovered through proteomics that iminostilbene reduces HIF-1α expression and STAT3 phosphorylation by targeting PKM2, mitigating inflammation and myocardial damage after MI. In addition, a recent study found that hypoxia promotes M2 macrophage intracellular HIF-1α expression and nuclear translocation. It binds to the VSIG4 promoter region, mediating its expression, thereby enhancing the secretion of IL-10 and TGF-β by macrophages, regulating cardiac fibroblast proliferation, migration, and phenotypic transition, and promoting myocardial repair after infarction [130]. Not only in MI but also in other cardiovascular diseases, this pathway has been shown to affect macrophage function. In apolipoprotein E-deficient mice, myeloid-specific loss of HIF-1α leads to restricted macrophage necrosis, reducing necrotic core formation and atherosclerosis progression. This effect is achieved by downregulating microRNA-210 (miR-210) and upregulating miR-383 in cells, promoting oxidative phosphorylation and ATP production, reducing mitochondrial reactive oxygen species accumulation, and inhibiting macrophage necroptosis [131]. Myocardial remodeling induced by the TAC model also leads to tissue hypoxia, promoting the recruitment of Ly6C^hi^ pro-inflammatory macrophages to the hypoxic area in an HIF-1α-dependent manner. These macrophage subpopulations secrete oncostatin-M, inhibiting TGF-β/SMAD signaling to deactivate cardiac fibroblasts, preventing excessive myocardial fibrosis [132].

Taken together, hypoxic microenvironment in MI drives cellular metabolic regulation, leading to different functional phenotypes of macrophages. Therefore, metabolic reprogramming may represent an innovative intervention target for future therapies. Zhao et al. [133] used the aspartate aminotransferase inhibitor amino-oxyacetic acid in vitro to suppress lactate production and glycolysis in M1 macrophages and increase ATP levels, thereby attenuating inflammasome pathway activation and reducing the secretion of pro-inflammatory cytokines. A recent study indicated that histone lactylation in bone marrow and circulating monocytes contributes to the construction of a reparative environment and improvement of cardiac function after MI reperfusion injury [134]. This is mainly achieved by promoting the early remote activation of reparative genes in monocyte-derived macrophages, thus regulating both the anti-inflammatory and pro-angiogenic effects. Mechanistically, monocytes undergo metabolic reprogramming in the early stage of MI and promote histone lactylation through dysregulated glycolysis and monocarboxylate transporter 1-mediated lactate transport. In the MI microenvironment, metabolic regulation occurs not only in monocytes/macrophages but also through cross-talk, influencing the interaction between different cell types. Lactate synthesis mediated by lactate dehydrogenase A (LDHA) contributes to the construction of a microenvironment favorable for myocardial regeneration. Cardiomyocyte-specific knockout or overexpression of LDHA affects cardiomyocyte proliferation and cardiac repair after MI, mainly due to reduced succinyl coenzyme A inhibition of thioredoxin reductase 1 ubiquitination mediated by LDHA, leading to decreased oxidative stress, promotion of cardiomyocyte proliferation, and even induction of M2 macrophage polarization [135].

### Neovascularization

Neovascularization in the body is tightly regulated, maintaining a delicate balance through the coordinated action of factors that promote and inhibit it [136]. Following MI, neovascularization primarily occurs in the infarct border, subendocardial interval, and epicardial regions, where newly formed microvessels emerge in mice. From the second to the fourth day after MI, the capillary network in the border zone gradually expands and branches until it reaches the infarct core. By the seventh day after MI, most endothelial cells in the border zone cease proliferating, while some newly formed capillaries enlarge and gain support from smooth muscle cells [137]. In various models, including MI, stroke, or skin injury, the number of macrophages positively correlates with the extent of neovascularization. This relationship may arise from the formation of new blood vessels that facilitate macrophage infiltration and expedite the clearance of necrotic cells. Furthermore, the improvement of the injury microenvironment further stimulates neovascularization [138]. Astragalus polysaccharides stimulate macrophages, inducing their polarization into the M2 phenotype, which in turn influences co-cultured endothelial cells, promoting their migration and neovascularization [139]. Activated macrophages, as key effectors in neovascularization, generate a plethora of vascular growth-related stimuli and inhibitory factors, including VEGF, IL-1, TNF-α, and TGF-β. These factors affect various stages of neovascularization, such as local ECM changes, induction of endothelial cell migration or proliferation, and capillary differentiation [137]. Liu et al. [140] observed that pro-inflammatory M1 macrophages release a large number of extracellular vesicles after MI, which transmit key components to endothelial cells and modulate their proliferation and migration by downregulating multiple target genes like RAC1, PAK2, and Sirt1. Ultimately, this exacerbates myocardial injury and impedes heart healing. Huang et al. [141] also confirmed the crucial role of macrophages in MI wound healing. Lgr4-specific knockout mice exhibited reduced myocardial cell apoptosis, decreased local myocardial inflammation levels, enhanced neovascularization, and deposition of reparative granulation tissue after MI. Similarly, genetic deletion of YAP/TAZ in macrophages induced polarization towards the reparative phenotype, leading to increased neovascularization in the myocardium and improved heart function following MI [142]. Recently, Reboll et al. [143] identified the cytokine meteorin-like protein (METRNL), derived from monocytes/macrophages, as a driving force for neovascularization after MI. METRNL selectively amplifies the endothelial cell subpopulation in the infarct border area through the stem cell factor receptor KIT-dependent signaling pathway, promoting repair after MI. METRNL-deficient mice did not exhibit this response and developed severe heart failure after MI. Ferraro et al. [144] discovered that knockout of annexin A1 (AnxA1) in mice with MI led to an expansion of pro-inflammatory macrophages in the infarct area and deterioration of cardiac function. AnxA1 knockout mice demonstrated significantly decreased VEGF secretion by cardiac macrophages, while exogenous administration of AnxA1 reversed this effect. Compared to non-diabetic mice, those with diabetes often experience more severe heart dysfunction after MI, possibly due to endothelial dysfunction in diabetic mice. Jiao et al. [145] observed a significant decrease in nicotinamide adenine dinucleotide (NAD^+^) levels in the heart tissue of mice with MI, particularly in those with diabetes. They also found that the administration of exogenous NAD could regulate macrophage M2 polarization, enhance VEGF cleavage, increase its secretion, promote endothelial cell migration and tube formation, thereby alleviating myocardial injury. These findings suggest that exogenous NAD may hold promise as a therapeutic target for MI in diabetic patients.

### Cardiac regeneration

As mentioned earlier, MI leads to irreversible damage to adult cardiomyocytes, rendering cell regeneration unfeasible. Consequently, myocardial wound healing relies on the immune system-mediated repair pathway. Following MI, activated immune cells migrate to the infarcted area, facilitating debris clearance and the formation of fibrotic scar tissue to repair the damaged region. The inflammatory and repair processes post-MI are relatively fixed and primarily regulated by the innate immune system. Although the initial inflammatory response is advantageous, scar formation can contribute to pathological cardiac remodeling and progressive impairment of cardiac function over time. Chronic inflammation resulting from the conversion of acute inflammation can cause sustained harm to the heart, hindering post-injury healing. In a typical MI, billions of cardiomyocytes are lost. Considering that adult human cardiomyocytes exhibit a low proliferation rate of approximately 1% per year, addressing this fundamental issue remains a challenge in cell therapy [146]. Notably, salamanders, zebrafish, and newborn mammals possess a natural mechanism for myocardial regeneration, which may be suppressed or regulated by external factors in adults [147, 148]. Research has demonstrated a negative correlation between immune system development and regenerative ability in lower vertebrates like African clawed frog larvae, underscoring the significance of immune cells in regulating regeneration [149]. Godwin et al. [150] discovered that macrophage infiltration is a prerequisite for limb regeneration in salamanders, as systemic macrophage depletion leads to wound closure without subsequent limb regeneration. Excessive expression of fibrosis and ECM component genes may contribute to this outcome, while replenishing the endogenous macrophage population can fully restore regenerative capacity at the amputation site. The team further revealed that macrophage-mediated regulation of fibroblast activation and ECM is also critical for salamander heart regeneration, although it does not affect cardiomyocyte proliferation [151]. In a zebrafish model of heart regeneration, Sanz-Morejon et al. [152] identified a subset of macrophages expressing wt1b that can be recruited to the regenerative area, exhibiting unique gene expression profiles and migration abilities that promote regeneration. The absence of such subsets results in reduced cardiomyocyte proliferation following heart injury and delayed growth after caudal fin amputation. More recently, Bruton et al. [153] discovered that macrophages are selectively recruited to the epicardium during juvenile zebrafish heart regeneration, promoting cardiomyocyte proliferation by increasing VEGF expression in the epicardium.

Interestingly, the maturation of the immune system aligns with the developmental timeline of myocardial cell terminal differentiation, indicating a potential influence of immunity on the cardiac regeneration ability of mammals. Neonatal mice possess a significant population of macrophages in their hearts, and it has been observed that macrophages accumulate briefly at the site of myocardial excision and subsequent regeneration in neonatal mouse hearts, suggesting a correlation between macrophages and cardiac regeneration [154]. Aurora et al. [155] conducted a comparative analysis of the immune response in postnatal day 1 (P1) mice (with regenerative ability) and P14 mice (with diminished regenerative ability) following MI and investigated differences in mononuclear/macrophage cell responses to injury. In P1 mice, macrophages exhibited greater abundance and uniform distribution throughout the heart after MI, whereas in P14 mice, macrophage numbers were lower and confined to the infarcted area after MI, with no significant variation in neutrophil presence between the two groups. Depletion of macrophages using clodronate liposomes prevented neonatal mice from regenerating their myocardium and impeded effective angiogenesis post-MI, resulting in impaired cardiac function and fibrotic scar formation. Transcriptome analysis revealed a distinct polarization phenotype of macrophages during the regeneration phase, with the ability to secrete multiple cytokines to promote cardiac regeneration. Building upon this, Lavine et al. [46] proposed that RCMs, rather than recruited macrophages, play a central role in mediating cardiac regeneration in neonatal mice. These embryonic-derived RCMs exhibit superior phagocytic, angiogenic, and reparative properties. Conversely, replacing repair-type RCMs with peripherally recruited inflammatory CCR2^+^ macrophages in adult mice after MI can lead to pathological remodeling and functional impairments. Li et al. [156] demonstrated that osteopontin serves as a crucial upstream regulatory factor for neonatal mouse myocardial cell proliferation. Macrophage-specific knock-out of osteopontin in neonatal mice inhibited cardiac regeneration following cardiac injury, highlighting the critical role of macrophage recruitment and osteopontin secretion in cardiac regeneration. Zlatanova et al. [157] discovered that ferritin inhibits macrophage-mediated cardiac repair after cardiac injury. In a neonatal mouse heart apex resection model, the absence of macrophage ferritin promoted myocardial cell proliferation, facilitating heart regeneration, potentially through STAT3 signal phosphorylation to regulate the secretion of IL-4/IL-13. Recent evidence suggests that transplanting sorted heart macrophages from neonatal mice, following heart apex resection, into adult mice after MI can promote heart healing by inducing adult myocardial cell proliferation [158]. Therefore, amidst the challenges faced in stem cell therapy, regulating the function of specific macrophage subgroups after acute MI to promote cardiac regeneration and repair holds promise as a future therapeutic approach.

## Macrophages-targeted translational research

### Extracellular vesicles-based therapy

Exosomes, recognized as crucial intercellular communication tools, play a vital role in various physiological and pathological processes [159]. With their lipid bilayer structure and ability to carry miRNA, proteins, and lipids, extracellular vesicles have garnered increasing attention in the field of regenerative medicine [160]. Mesenchymal stem cells (MSCs) have been widely employed for the treatment of ischemic heart disease; however, their underlying mechanism remains incompletely understood. Zhao et al. [161] conducted an experiment in which MSC-derived exosome (MSC-Exo) was administered to mice after reperfusion injury, resulting in targeted inhibition of toll-like receptor 4 (TLR4) in macrophages through miR-182 delivery. This inhibition promoted the transformation of M1-like macrophages into the M2-like phenotype, ultimately reducing the infarcted area and alleviating inflammation levels in the heart and serum. Similarly, by utilizing MSC-Exo loaded with miR-101a as a biologically active carrier for heart repair, macrophages can be induced to adopt an anti-inflammatory phenotype, thereby improving heart function post-MI [162]. Furthermore, when pro-inflammatory MSCs were stimulated with low-concentration lipopolysaccharides, the released MSC-Exo effectively mediated macrophage polarization towards the M2-like phenotype, resulting in reduced inflammation and apoptosis of myocardial cells following infarction [163]. MSC-Exo derived from MSCs overexpressing FNDC5 demonstrated superior anti-inflammatory and anti-apoptotic effects compared to normal MSC-Exo, facilitating macrophage polarization towards the M2 direction [164]. Deng et al. [165] extracted MSC-Exo from adipose-derived MSCs and demonstrated their ability to promote macrophage M2 polarization through activation of the S1P/SK1/S1PR1 pathway, ultimately alleviating myocardial injury after MI. In addition to MSCs, de Couto et al. [166] discovered that exosomes secreted by cardiosphere-derived cells (CDCs), but not fibroblasts, can decrease the number of CD68^+^ macrophages in the infarcted area following reperfusion injury in rabbits and pigs. These exosomes were also able to modulate macrophage polarization phenotype by delivering miR-181b. Notably, CDC-Exo exhibited a preference for M1-like macrophages and upregulated Arg-1 expression, promoting a pro-angiogenic phenotype and downregulating iNOS secretion [167]. Recently, it has been revealed that exosomes derived from ferroptotic cardiomyocytes and dendritic cell-loaded hydrogels can respectively regulate macrophage polarization towards M1 and M2 types after MI [168, 169]. Moreover, even macrophage-secreted extracellular vesicles contribute to the regulation of the inflammatory repair process in cardiac injury through intercellular communication [170]. Wang et al. [171] observed elevated miR-155 expression in the hearts of mice following MI, primarily in macrophages and fibroblasts. Macrophages transmitted miR-155 to fibroblasts via extracellular vesicles, thereby inhibiting cardiac fibroblast proliferation and promoting inflammation. This effect was reversed by a miR-155 inhibitor, suggesting its potential as a therapeutic agent for reducing adverse events post-MI. Furthermore, Long et al. [172] confirmed that extracellular vesicles derived from M2 macrophages can mitigate myocardial cell apoptosis and alleviate heart injury after MI by delivering miR-1271-5p and downregulating SOX6 expression. Nevertheless, the limited retention and short-term effectiveness of extracellular vesicles necessitate the engineering modification of these vesicles and the prevention of macrophage internalization, which can reduce off-target effects and enhance their delivery efficiency to target cells. Various techniques can be employed for this purpose, including the use of platelet-derived nanovesicles, monocyte-mimicking modification, and alginate hydrogel [173–175].

### Nanomaterial-mediated drug delivery therapy

Specially modified nanocarriers, such as liposomes and polymer nanoparticles, have found extensive applications in cardiovascular and tumor treatments, enhancing the transport efficiency of therapeutic drugs [176]. These nanocarriers, loaded with small interfering RNA or drugs, effectively mediate gene expression in macrophages after MI and induce their polarization towards the M2 phenotype, thereby promoting heart repair. Liposomes, similar to extracellular vesicles, are lipid bilayer-based drug delivery systems with diameters ranging from hundreds to thousands of nanometers [177]. Encapsulation of berberine within liposomes enhances its solubility in buffer solutions, effectively suppressing IL-6 secretion in macrophages and preserving heart function following MI [178]. Subcutaneous administration of liposomal nanoparticles, loaded with MI antigens and rapamycin, in MI mice, mitigates cardiac inflammation, and inhibits ventricular remodeling by modulating antigen-specific T cell regulation and promoting M2-like macrophage polarization [179]. Liposomal delivery of azithromycin also significantly reduces cardiac toxicity and related mortality. The formulation aids in decreasing neutrophil and monocyte infiltration post-MI, upregulating repair-related genes in macrophages, and preventing off-target effects [180]. Bao et al. [181] proposed a biomimetic strategy involving engineered neutrophil apoptotic bodies combined with mesoporous silica nanoparticles carrying hydrochloride. This approach effectively targets macrophages and initiates the biosynthesis pathway of anti-inflammatory heme, thus regulating macrophage function and promoting MI repair. Similarly, Li et al. [182] employed mesoporous silica nanoparticles encapsulating miR-21-5p within an injectable hydrogel. This system enables precise drug delivery triggered by the local acidic environment, inhibits M1-like macrophage polarization in the infarcted myocardium, and promotes local blood vessel neogenesis. Leveraging the nanocarrier platform, drugs like pioglitazone or TLR4 inhibitors can be efficiently delivered to monocytes/macrophages in the spleen, blood, and heart. This approach counteracts acute inflammatory responses, facilitating improved myocardial repair after MI in mice or pigs [183, 184].

### Precise intervention against macrophages: a challenge for the future

**Fig. 4:**
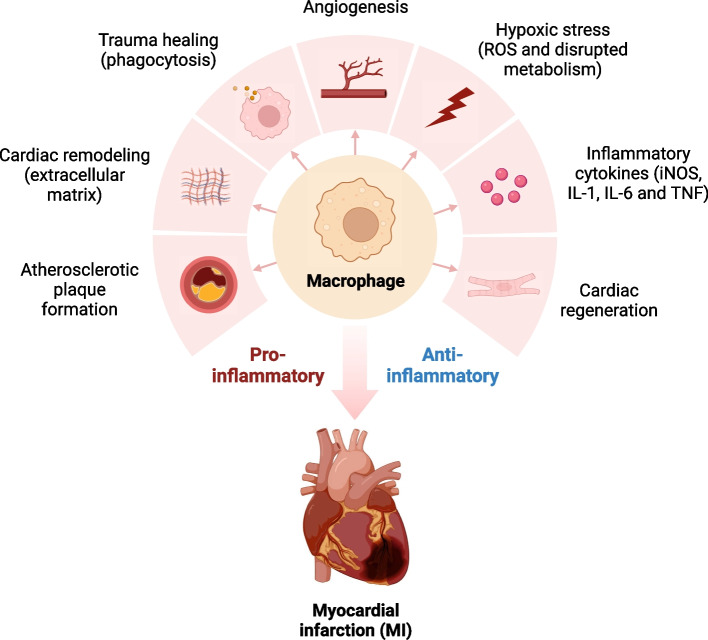
The role of macrophages in inflammation and repair after MI. In summary, macrophages play several key roles in myocardial infarction (MI): from the initial formation of atherosclerosis to stress-induced damage during the acute inflammatory phase, secretion of pro-inflammatory factors, and finally their involvement in the phagocytosis of necrotic myocardial cells, synthesis of the extracellular matrix, angiogenesis, and myocardial regeneration during the repair phase. The pro-inflammatory and anti-inflammatory effects mediated by macrophages are intertwined throughout the development of MI, and they engage in cross-talk with myocardial cells, cardiac fibroblasts, and endothelial cells. Targeting macrophage-mediated inflammation regulation is a promising clinical strategy for the effective treatment of MI in the future. Adapted from “Mechanisms of Cancer-associated Fibroblast Activation”, by BioRender.com (2020). Retrieved from https://app.biorender.com/biorender-templates

After MI, macrophages not only function as effectors but also influence other cells within the ischemic myocardium through paracrine mechanisms, thereby affecting the recovery of cardiac function after MI (Fig. 4). Considering the significance of cardiac macrophages in cardiac injury and remodeling after MI, conducting transformative research on cardiac macrophages appears to be highly promising. However, before clinical application, there remain numerous unresolved challenges. It is not only the high heterogeneity and plasticity of macrophages that pose obstacles to precise interventions but also the potential for poor survival rates of transplanted embryonic-origin CRM due to immune system rejection. Fortunately, cell-free therapy offers a new perspective, with extracellular vesicles and nanomaterial drug delivery systems expected to become powerful tools for targeting macrophage regulation after MI in the future. Currently, research targeting macrophages mostly focuses on regulating macrophage polarization and their levels of inflammation, lacking more precise therapeutic strategies targeting macrophage subtypes identified through single-cell sequencing in the era. As our understanding of the functional mechanisms of macrophages after MI deepens, we believe that precise interventions distinct from traditional broad anti-inflammatory treatment regimens will emerge. For example, a recent study isolated extracellular vesicles produced by CCR2^+^ cardiac macrophages, combined with monocyte membrane modification and nanoparticle-loaded thymosin β4 [185]. This enhanced their targeting precision and anti-immune rejection characteristics, ultimately mimicking the physiological effects achievable by CCR2^+^ cardiac macrophages, including promoting myocardial cell proliferation and angiogenesis. Additionally, Li et al. [186] used small extracellular vesicles derived from M2 macrophages to regulate glycolysis and mitochondrial reactive oxygen species production in CCR2^+^ macrophages after MI through miR-181b-5p, thereby improving CCR2^+^ macrophage infiltration and infarct size.

## Summary and prospect

Recent findings have unveiled new targets for intervention to regulate cardiac inflammation and repair, providing a scientific foundation for further in-depth investigations. Diverse treatment modalities, including hydrogels, nanoparticles, and extracellular vesicles, have shown promise in enhancing post-MI healing, myocardial repair, and regeneration by modulating macrophage function. Notably, with the rapid advancement of emerging technologies like scRNA-seq, the scientific community's comprehension of macrophage subgroups in the heart continues to deepen, fostering the potential for targeted therapies focused on specific mononuclear cells or macrophage subgroups. These approaches hold the potential to optimize cardiac inflammatory responses and facilitate myocardial regeneration following MI.

To effectively translate these scientific discoveries into clinical applications and bridge the gap between laboratory and clinic, further research is warranted to gain a comprehensive understanding of macrophage regulation of myocardial regeneration and repair in neonatal and adult individuals at different stages of cardiac injury. As underscored in this review, macrophages exhibit considerable plasticity and heterogeneity, and their roles in cardiac repair and regeneration are intricate, necessitating additional investigations to elucidate underlying mechanisms. It is worth noting that while the neonatal mouse heart apex resection model may not precisely mimic the inflammation and repair processes after MI, gaining insights into the interactions between macrophages and myocardial cells, fibroblasts, and endothelial cells in neonatal mice remains crucial for developing future therapies. Particularly, embryonic-derived CCR2^−^ macrophages hold potential in improving clinical outcomes for adult patients with irreversible myocardial injury. Furthermore, these strategies may also find applicability in other inflammation-associated cardiovascular diseases, such as atherosclerosis and myocarditis. Additionally, despite the significant improvements in drug delivery systems, which have enhanced the efficiency of therapeutic drug application, several challenges, including safety and specificity, necessitate further exploration and verification prior to practical implementation.

## Data Availability

No data was used for the research described in the article.

## Abbreviations

MI: Myocardial infarction
RTMs: Resident tissue macrophages
ECM: Extracellular matrix
MMPs: Matrix metalloproteinases
IL: Interleukin
RNA: Ribonucleic acid
scRNA-seq: Single-cell ribonucleic acid sequencing
CRMs: Cardiac resident macrophages
CCR2: CC chemokine receptor 2
MHC II: Major histocompatibility complex II
Timd4: T-cell immunoglobulin and mucin domain-containing protein 4
Cx43: Connexin 43
Mertk: C-mer tyrosine kinase
TNF-α: Tumor necrosis factor-α
Trem2: Triggering receptor expressed on myeloid cells 2
iNOS: Inducible nitric oxide synthase
FPR2: Formyl peptide receptor 2
VEGF: Vascular endothelial growth factor
TGF-β: Transforming growth factor-β
HIFs: Hypoxia-inducible factors
PPP: Pentose phosphate pathway
TCA: Tricarboxylic acid
ATP: Adenosine triphosphate
PKM2: Pyruvate kinase M2
LDHA: Lactate dehydrogenase A
METRNL: Meteorin-like protein
AnxA1: Annexin A1
NAD+: Nicotinamide adenine dinucleotide
MSC: Mesenchymal stem cell
MSC-Exo: Mesenchymal stem cell-derived exosome
CDCs: Cardiosphere-derived cells
TLR4: Toll-like receptor 4
